# Loss of presenilin function enhances tau phosphorylation and aggregation in mice

**DOI:** 10.1186/s40478-021-01259-7

**Published:** 2021-09-30

**Authors:** Carlos M. Soto-Faguás, Paula Sanchez-Molina, Carlos A. Saura

**Affiliations:** 1 Institut de Neurociències, Universitat Autònoma de Barcelona, 08193 Barcelona, Spain; 2 Departament de Bioquímica i Biologia Molecular, Facultat de Medicina, Universitat Autònoma de Barcelona, 08193 Barcelona, Spain; 3 Departament de Biologia Cel·lular, Fisiologia i Immunologia, Facultat de Medicina, Universitat Autònoma de Barcelona, 08193 Barcelona, Spain; 4 Centro de Investigación Biomédica en Red Enfermedades Neurodegenerativas (CIBERNED), Instituto de Salud Carlos III (ISCIII), Madrid, Spain

**Keywords:** Alzheimer’s disease, Memory, Neurofilament, Presenilin, γ-secretase, Tau, Synapse, β-sheet aggregation

## Abstract

Mutations in the presenilin (*PS/PSEN*) genes encoding the catalytic components of γ-secretase accelerate amyloid-β (Aβ) and tau pathologies in familial Alzheimer’s disease (AD). Although the mechanisms by which these mutations affect Aβ are well defined, the precise role PS/γ-secretase on tau pathology in neurodegeneration independently of Aβ is largely unclear. Here we report that neuronal *PS* deficiency in conditional knockout (cKO) mice results in age-dependent brain atrophy, inflammatory responses and accumulation of pathological tau in neurons and glial cells. Interestingly, genetic inactivation of *presenilin 1* (*PS1*) or both *PS* genes in mutant human Tau transgenic mice exacerbates memory deficits by accelerating phosphorylation and aggregation of tau in excitatory neurons of vulnerable AD brain regions (e.g., hippocampus, cortex and amygdala). Remarkably, neurofilament (NF) light chain (NF-L) and phosphorylated NF are abnormally accumulated in the brain of Tau mice lacking PS. Synchrotron infrared microspectroscopy revealed aggregated and oligomeric β-sheet structures in amyloid plaque-free PS-deficient Tau mice. Hippocampal-dependent memory deficits are associated with synaptic tau accumulation and reduction of pre- and post-synaptic proteins in Tau mice. Thus, partial loss of PS/γ-secretase in neurons results in temporal- and spatial-dependent tau aggregation associated with memory deficits and neurodegeneration. Our findings show that tau phosphorylation and aggregation are key pathological processes that may underlie neurodegeneration caused by familial AD-linked *PSEN* mutations.

**Supplementary Information:**

The online version contains supplementary material available at 10.1186/s40478-021-01259-7.

## Introduction

Alzheimer’s disease (AD) is the most prevalent and disabling memory disorder in the elderly. AD is characterized by neurodegeneration, brain inflammation and accumulation of amyloid-β (Aβ) plaques and neurofibrillary tangles (NFTs) containing bundles of paired helical filaments (PHF) formed of aggregated hyperphosphorylated microtubule-associated protein tau (MAPT) in memory brain regions [23, 62]. Phosphorylation and aggregation of tau are also prevalent pathological features of other tauopathies, including frontotemporal dementias (FTD), corticobasal degeneration (CBD), Pick’s disease and progressive supranuclear palsy (PSP) [23]. Recent studies suggest that trans-synaptic spreading of pathological tau among neural circuits is critical in tauopathies [23, 42], although the underlying mechanisms are still largely unclear. In AD, cognitive decline correlates with progression of tau pathology rather than amyloid plaque deposition in limbic regions [1, 26]. Aβ and tau act synergistically to induce synapse dysfunction [32, 49], a pathological feature tightly associated with early memory loss [43, 59, 66]. Pathological tau is present at synapses and induces synapse dysfunction, instability and loss [29, 33, 73]. Whether synaptic tau is responsible of synapse pathology and behavioral changes in AD is unclear.

Autosomal dominant mutations in the *presenilin* (*PSEN*/*PS*) genes encoding presenilin 1 (PS1) and presenilin 2 (PS2) cause early-onset familial AD presumably by accelerating Aβ and/or tau pathologies [13]. PS are the catalytic components of γ-secretase that cleaves the amyloid precursor protein (APP) to generate Aβ, and when mutated increase amyloidogenic Aβ species and tau-associated neurites and NFTs [25, 63, 71]. PS1 regulates neurofilament (NF) assembly and neurite extension, whereas mutant PS1 causes cytoskeletal changes associated with increased tau phosphorylation, release from microtubules and binding to NF [19, 50, 71]. PS1 mutations are also linked with tau aggregation, in the presence or absence of Aβ pathology, in the frontal cortex of FTD [4, 16, 54], suggesting that PS dysfunction causes tau pathology independently of Aβ. Consistent with a role of PS on tau phosphorylation, AD-linked PS1 mutations increase GSK3β activation, tau phosphorylation and amyloid deposition, and cause synaptic dysfunction and neurodegeneration in *knockin* mice [17, 72]. Considering that familial AD-linked PS mutations interfere with γ/ε-secretase cleavage and biological functions [40], a loss-of-function mechanism for PS mutations has been proposed [60]. In agreement, loss of neuronal PS results in age-dependent neurodegeneration associated with tau phosphorylation in *PS* conditional knockout (cKO) mice [46, 57, 70]. Whereas these studies clearly suggest that loss of PS/γ-secretase recapitulates key features of tauopathies, whether PS/γ-secretase regulates tau aggregation independently of Aβ is unknown.

Here, we investigated the role of PS in tau pathology by examining in vivo the effects of partial or total loss of PS in postmitotic neurons of novel tauopathy mouse models expressing a FTD-linked human *Tau* and lacking conditionally PS1 (PS1 cKO;Tau) or both PS (PS cKO;Tau) genes. We found that partial *PS* inactivation in Tau transgenic mice results in accelerated phosphorylation and aggregation of tau and NF light chain (NF-L) in memory neural circuits coinciding with loss of synaptic proteins and exacerbated memory deficits.

## Materials and methods

### Mice

PS cKO mice (C57BL/6/129 background) lacking *PS2* embryonically and *PS1* specifically in forebrain glutamatergic neurons were previously described [57]. Littermate control (*PS1* f/f; *PS2*^+/+^ or *PS1* f/f; PS2^+/-^; f: floxed), PS1 cKO (*PS1* f/f; CamKIIα-Cre) and PS cKO (*PS1* f/f; *PS2*^−/^; CaMKIIα-Cre) mice were obtained by crossing floxed PS1/PS2-/- (*PS1* f/f; *PS2*^−/−^) or PS2^±^ (*PS1* f/f; *PS2*^+/-^) males to heterozygous PS1 cKO; PS2^±^ females (*PS1* f/f; *PS2*^+/-^; CaMKIIα-Cre). We generated novel non-transgenic control (*PS1* f/f), tau (*PS1* f/f;Tau), PS1 cKO;Tau (*PS1* f/f;CamKIIα-Cre;Tau) and PS cKO;Tau (*PS1* f/f;*PS2*^−/−^;CamKIIα-Cre;Tau) mice by crossing PS1 cKO or PS cKO with Tau P301S transgenic mice (PS19; JAX #008169; C57BL/6). Tau P301S mice express the FTD-linked P301S Tau under the neuron-specific prion protein promoter [73]. All experimental procedures were conducted according to approved protocols from the Animal and Human Ethical Committee of the Universitat Autònoma de Barcelona (CEEAH 2895) and Generalitat de Catalunya (DMAH 10571) following the experimental European Union guidelines and regulations (2010/63/EU).

### Behavioral tests

Tail suspension test was performed by suspending the mouse from the tail for 5 min. Measures of movement of hind/forelimbs and clasping behavior are measured according to the severity of the hindlimb clasping (0: no clasping; 1–4: discontinuous clasping of one-four paws; 5: complete clasping of all paws usually with turns). Hippocampus-dependent spatial memory was examined in the Morris water maze test as described [20]. Mice were trained the first day with a visual cue (6 trials; maximum 60 s/trial) followed by 5-day training in the hidden-platform version of the Morris water maze. At day 6, a 60 s probe trial was performed. Time to reach the platform (escape latency), distance traveled (path length), swimming speed, and crossings and percentage of target quadrant occupancy were recorded and quantified using ANY-maze behavioral tracking software.

For contextual fear conditioning, mice handled for three days (3 min/day) were placed in a conditioning chamber (15.9 × 14 × 12.7 cm; Med Associates, St. Albans, Vermont) for 3 min, foot-shocked (1 s/1 mA) and retained in the chamber for 2 min (immediate freezing) [20]. Fear memory was tested as freezing behavior, defined as a complete cessation of all movement except for respiration, in the same conditioning chamber for 4 min 24 h after training using Video Freeze Software (Med Associates).

### Biochemical analysis and subcellular fractionation

For biochemical analysis, half hippocampus was lysed in cold-lysis buffer (62.5 mM Tris hydrochloride, pH 6.8, 10% glycerol, 5% β-mercaptoethanol, 2.3% sodium dodecyl sulfate [SDS], 5 mM NaF, 100 µM Na_3_VO_4_, 1 mM EDTA, 1 mM ethylene glycol tetraacetic acid) containing protease and phosphatase inhibitors and boiled at 100 °C [51, 67]. Synaptosome fractionation was performed essentially as described [58]. Individual mouse hippocampus was homogenized with a Teflon-glass homogenizer in ice-cold Buffer A (5 mM HEPES, pH 7.4, 0.32 M sucrose, 1 mM NaHCO_3_, 1 mM MgCl_2_, 0.5 mM CaCl_2_, 1 mM NaF and phosphatase and protease inhibitors). The homogenate was centrifuged (1,400 × *g*, 10 min) and the pellet (P1) was re-extracted with Buffer A and centrifuged (710 × *g*, 10 min). The supernatants were combined and centrifuged (13,800 × *g* for 10 min). The pellet was resuspended in ice-cold Buffer B (6 mM Tris, pH 8.0, 0.32 M sucrose, 1 mM NaHCO_3,_ 1 mM NaF and phosphatase and protease inhibitors) and loaded onto the top of a 0.85 M, 1 M and 1.2 M discontinuous sucrose gradient and centrifuged (82,500 × *g*, 2 h). The synaptosome fraction, collected from the 1 M–1.2 M sucrose interface, was diluted in equal volume of Buffer C (12 mM Tris HCl, pH 8.0, 1% Triton X-100). The suspension was spin at 32,800 × *g* for 1 h to obtain the presynaptic (supernatant) and the postsynaptic (pellet) fractions, the latter was resuspended in cold-ice Buffer D (40 mM Tris, pH 8.0, 1% NP40). Proteins were quantified using the protein assay kit (Invitrogen), resolved on SDS–polyacrylamide gel electrophoresis and detected by Western blotting using tau and NF antibodies (Table 1), and rabbit anti-GFAP (Dako, Z0334), Iba1 (Wako, 019–19741), PSD95 (Cell Signaling, 2507) and CREB-regulated transcription coactivator 1 (CRTC1, Cell Signaling, 2587) and mouse anti-synaptophysin (Sigma, S5768), syntaxin 1 (SantaCruz, sc-12736), β-tubulin (Sigma, T9026), β-actin (Sigma, A5441) and GAPDH (Life Technologies, AM4300) antibodies. Bands detected with secondary antibodies coupled to peroxidase (Bio-Rad) and enhanced chemiluminescent reagent were captured in ChemiDoc MP System (Bio-Rad) and quantified in a linear range with the ImageLab 5.2.1 software (Bio-Rad). Blot images were not spliced, and stripped and reblotted membranes are specified in the figure legends.

**Table 1:** Tau and NF antibodies used in this study

Name	Specificity	Dilution (WB/IH^a^)	Source / Reference
AT8	pSer202/Thr205 Tau	1:200	LifeTechnologies (BR-00390206)
CP13	pSer202 Tau	1:250/1:50	P. Davies/[34]
D1M9X	Tau	1:250	Cell Signaling (46687)
MC1	Conform. Tau (312–322)	1:250/1:50	P. Davies
NF-L	NF light chain	1:1,000/1:800	Merck-Millipore (AB9586)
PG5	pSer409 Tau	1:250/1:100	P. Davies/[34]
PHF-1	pSer396/404	1:250/1:200	P. Davies/[27]
SMI132	pNF-M/H	1:500/1:500	Biolegend (837904)
Tau17025	Tau	1:5,000	V. Lee
TG5	Tau	1:500	P. Davies/[34]

^a^*WB* Western blotting. *IH* Immunohistochemistry

### Immunohistochemistry

Mice were perfused transcardially with PBS and fixed in 4% phosphate-buffered paraformaldehyde before paraffin embedding. Coronal brain Sections (5 μm) were deparaffinized in xylene, rehydrated and microwave heated in citrate buffer (10 mM, pH 6.0). Sections were incubated overnight at 4 °C with the indicated primary antibodies (Table 1). For immunoperoxidase staining, sections were incubated with a biotin-conjugated anti-mouse secondary antibody (1:200) and revealed with the DAB peroxidase substrate kit (Vector laboratories) before imaging with a Nikon Eclipse 80i microscope. For double immunostaining, sections were incubated with mouse anti-phosphorylated tau (CP13) and rabbit anti- NeuN (Chemicon, ABN78), Iba1 (Wako, 019–19741), Olig2 (Milipore, AB9610) or GFAP (Dako, Z0334) antibodies overnight followed by a 90 min incubation with AlexaFluor-488/594-conjugated goat IgGs (1:400) and Hoechst (1:10,000; Thermo Fisher Scientific). Confocal images (40x; zoom 1 or 2) were obtained with a Zeiss Axio Examiner D1 LSM700 laser scanning microscope (Carl Zeiss Microcopy, Jena, Germany). All images were analyzed with ImageJ software.

### Congo red staining

For Congo Red staining we used the Amyloid Stain Congo Red (Sigma-Aldrich). Briefly, after deparaffinization and hydration, slides were stained in Mayer’s hematoxylin solution (Sigma-Aldrich) for 10 min and rinsed in tap water. Slides were placed in alkaline NaCl and stained with Alkaline Congo Red Solution (20 min). Samples were rinsed (× 3) in 100% ethanol, cleared in xylene, mounted in DPX Mountant (Sigma-Aldrich) and observed in Nikon Eclipse 90i microscope.

### Synchrotron-based Fourier transform infrared microspectroscopy (μFTIR) data acquisition

Mice were intracardially perfused for 10 min with 4% paraformaldehyde in 0.1 M Tris buffer (pH 7.4), brains were post-fixed in the same solution for 4 h at 4 °C, cryoprotected with 30% sucrose solution in 0.1 M Tris buffer for 48 h at 4 °C, and frozen in ice-cold 2-methylbutane (Sigma-Aldrich). Coronal Sections (8 μm) were cut on a cryostat (CM3050S Leica) and mounted onto polished CaF_2_ optical windows (CAFP20-1, Crystran, U.K). μFTIR study was carried out at the MIRAS beamline at ALBA Synchrotron Light Source (Barcelona, Spain) using a Hyperion 3000 microscope equipped with a 36 × magnification objective coupled to a Vertex 70 spectrometer (Bruker) and a mercury cadmium telluride (MCT) detector. Spectra acquisition and analysis were performed as reported [55]. Briefly, the spectra collection was done in transmission mode at 4 cm^−1^ spectral resolution, 10 μm × 10 μm aperture dimensions, and 128 scans. The measuring range was 4000–600 cm^−1^ and zero filling was performed with fast Fourier transform. Background spectra were collected from a clean area of each CaF_2_ window every 10 min. For each animal, a total of 100 spectra per cerebral region with a step size of 30 μm × 30 μm were acquired in the retrosplenial cortex and the corpus callosum. The spectra were obtained by means of Opus 7.5 software (Bruker).

### Synchrotron-based μFTIR spectra analysis

Unscrambler X software (CAMO Software) was used for data processing. Second spectra derivative was calculated using a Savitsky-Golay algorithm with an eleven-point smoothing filter and a polynomial order of 3. Intramolecular β-sheet and α-helix protein secondary structures were detected by infrared absorptions at 1635 cm^−1^ and 1656 cm^−1^, respectively. Intermolecular β-sheet structures corresponding to β-sheet aggregates absorb at 1625 cm^−1^. Antiparallel β-sheets corresponding to oligomeric β-aggregates were detected at 1695 cm^−1^. In addition, protein/lipid (Amide I/CH_2_) and lipid oxidation, determined by carbonyl content (C=O) absorbances at 1741 cm^−1^ and the unsaturated olefinic group (C=CH) at 3014 cm^−1^, were measured to provide biochemical composition of the brain tissue. To account for variations in the tissue thickness, absorbances values were normalized by the major lipid content (CH_2_) or the major protein content (Amide I), giving rise to the following ratios: β-sheet/α-helix (d^2^A_1635_/d^2^A_1656_), β-intermolecular/Amide I (d^2^A_1625_/d^2^A_1635+1656_), β-antiparallel/Amide I (d^2^A_1695_/d^2^A_1635+1656_), Amide I/CH_2_ (d^2^A_1635+1656_/d^2^A_2921_), C=O/CH_2_ (d^2^A_1741_/d^2^A_2921_) and C=CH/CH_2_ (d^2^A_3014_/d^2^A_2921_).

### Statistical analysis

Statistical analysis (Prism software, GraphPad, La Jolla, CA) was performed using one or two-way Analysis of Variance (ANOVA) followed by Sidak’s (age comparison) or Tukey’s post hoc test for multiple comparisons and *t*-test when only two groups were compared. *P* values less than 0.05 were considered significant. The significance level is indicated using asterisks: * *P* < 0.05, ** *P* < 0.01, *** *P* < 0.001 and **** *P* < 0.0001.

## Results

### Loss of PS/γ-secretase function results in age-dependent neurodegeneration and tau phosphorylation

**Fig. 1:**
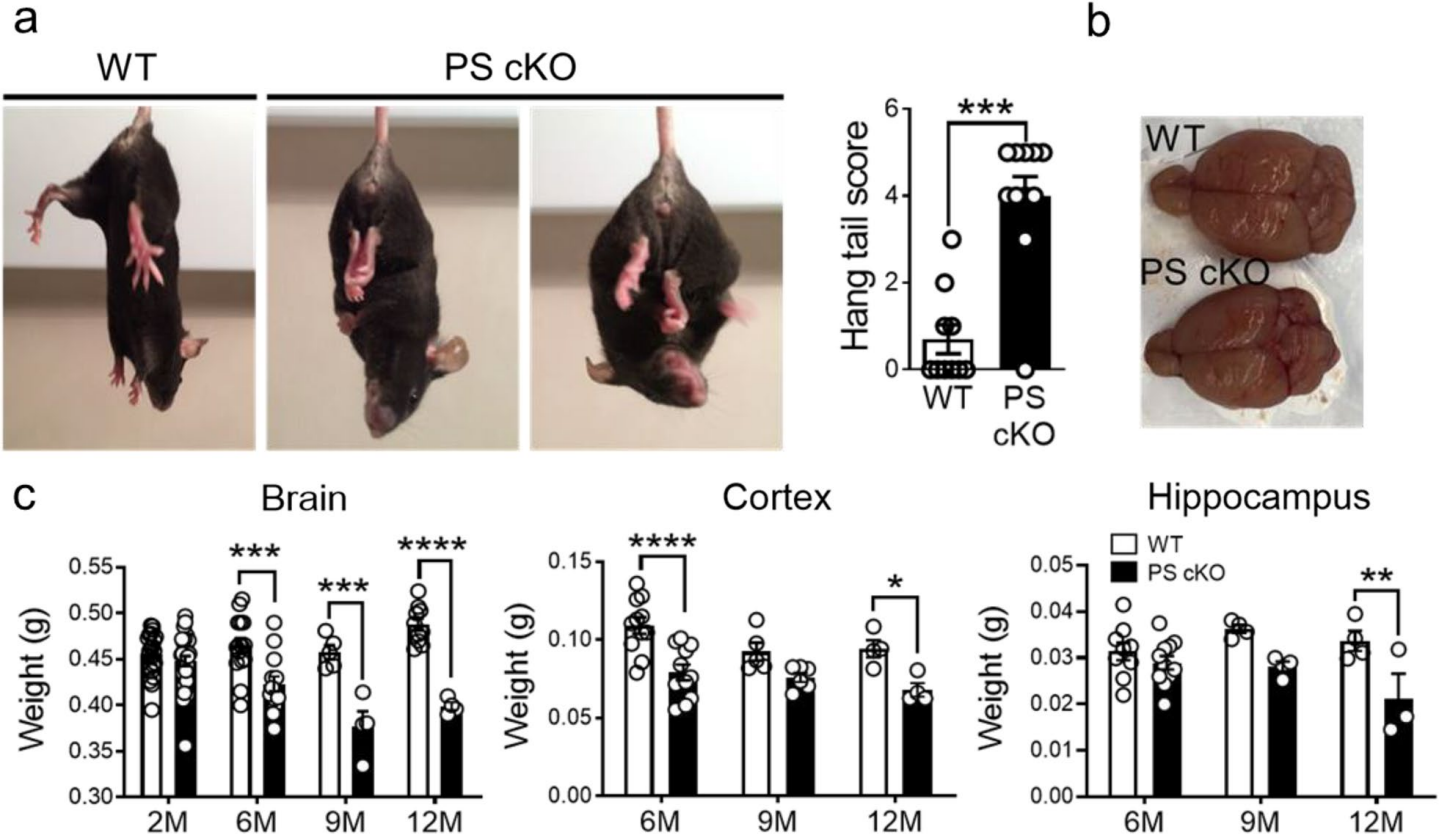
Loss of PS causes behavioral abnormalities and age-dependent neurodegeneration in mice. **a** Clasping and postural alterations in PS cKO mice at 9 months of age. Tail suspension test reveals that PS cKO mice show severe clasping behavior, as observed in PS cKO mice scoring 4 (left image) and 5 (right picture). Quantification graph: Statistical differences in hang tail score in PS cKO mice at 9 months. **b** Decreased brain size, especially the cortical hemispheres in PS cKO mice. **c** Age-dependent brain atrophy in PS cKO mice. Total brain (left), cortex (middle) and hippocampus (right) weights are reduced in PS cKO mice during aging (2 to 12 months). Values represent mean ± s.e.m (n = 4–30 mice/group). Statistical analysis was determined by two-way ANOVA followed by Sidak’s post hoc tests. *P < 0.05, ** *P* < 0.01 and ***P < 0.001 compared to control (WT) mice

To elucidate the role of neuronal PS/γ-secretase on tau phosphorylation during neurodegeneration, we performed behavioral, pathological and biochemical analysis in control (WT: PS1 f/f) and PS1 cKO;PS2^−/−^ (PS cKO: PS1 f/f;PS2^−/−^; CamKIIα-Cre) mice, in which both *PS* are deleted in excitatory neurons of the postnatal forebrain [57]. PS cKO mice at 6–9 months of age show behavioral alterations, including stereotypic, postural and clasping phenotypes (Fig. 1a). Tail suspension test revealed that PS cKO mice did not adopt a splayed limb position and showed elevated clasping scores indicative of motor deficits (*P* < 0.0001). Interestingly, weight of the whole brain, cortex and hippocampus are significantly reduced in PS cKO mice at 6–12 months of age (Two-way ANOVA, brain: genotype effect, *P* < 0.0001; age effect, *P* < 0.01; interaction effect, *P* < 0.0001; cortex: genotype effect, *P* < 0.0001; age effect, *P* < 0.05; hippocampus: genotype effect, *P* < 0.05; Fig. 1b, c). These results confirmed that, in contrast to PS1 cKO mice, PS cKO mice develop brain atrophy and features of neurodegeneration during aging [57, 70].

**Fig. 2:**
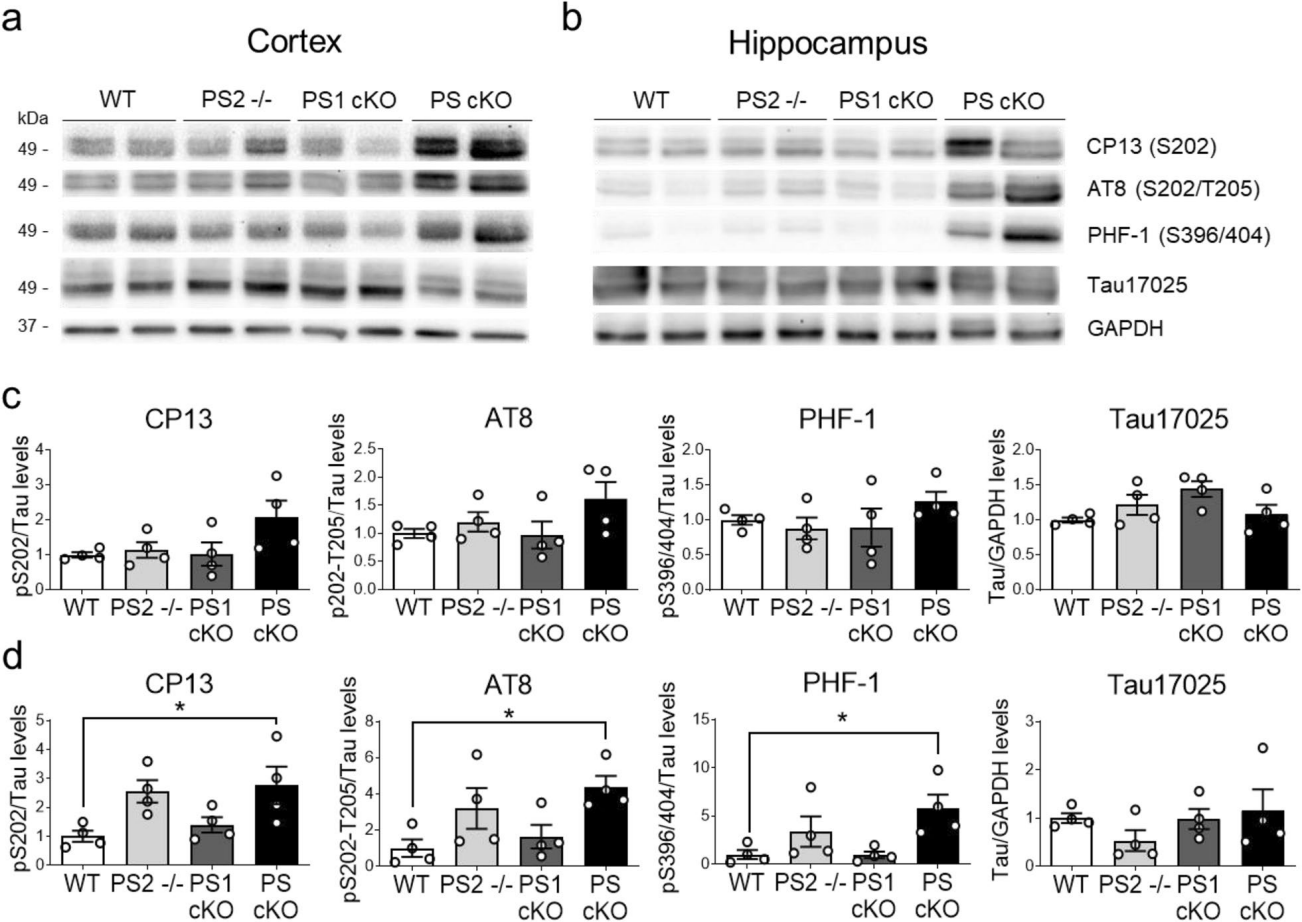
Dose-dependent presenilin effects on tau phosphorylation**.** Biochemical analysis showing increased tau phosphorylation in the cortex and hippocampus of PS cKO mice. Western blot images (top) and quantitative analysis (bottom) of protein lysates of cortex (**a, c**) and hippocampus (**b, d**) of WT, PS2^−/−^, PS1 cKO and PS cKO mice at 9 months of age. Western blotting was performed using antibodies against total tau (Tau17025) and anti-phosphorylated tau: Ser202 (CP13), Ser202/Thr205 (AT8) and Ser396/404 (PHF-1) tau. Phosphorylated tau levels were normalized to total tau. Tau17025 was incubated in the CP13 membrane after stripping. Values represent mean ± s.e.m. (n = 4 mice/group). Statistical analysis was determined by one-way ANOVA followed by Tukey’s post hoc tests. *P < 0.05

To examine whether neurodegeneration caused by loss of PS/γ-secretase was accompanied by tau pathology, we next performed biochemical analysis of endogenous murine tau in brains of control, PS2^−/−^, PS1 cKO, and PS cKO mice at 9 months of age. Biochemical analysis using anti-tau antibodies for phosphorylated Ser 202 (CP13) and Ser 202/Thr205 (AT8) present in early (pre-tangles) and advanced NFTs, and Ser 396/404 (PHF-1), a marker of late NFT stages in AD brain (Table 1), revealed enhanced tau phosphorylation in the cortex and hippocampus of PS cKO mice (Fig. 2a, b). Statistical analysis showed significant genotype effects for CP13, AT8 and PHF-1 in the hippocampus (*P* < 0.05), but not in the cortex, whereas post-hoc analysis revealed enhanced phosphorylated tau levels in PS cKO mice (Fig. 2c,d). Quantification of total tau using Tau17025 antibody revealed similar total tau (fold change in cortex/hippocampus) in control (1 ± 0.04/1 ± 0.11), PS2^−/−^ (1.22 ± 0.15/0.54 ± 0.22), PS1 cKO (1.44 ± 0.11/0.98 ± 0.21), and PS cKO (1.08 ± 0.14/1.15 ± 0.44) mice (*P* > 0.05; Fig. 2a, b). These results indicate that PS reduces endogenous tau phosphorylation in a gene dosage-dependent manner.

### Region- and cell-specific accumulation of phosphorylated tau in PS-deficient mice

**Fig. 3:**
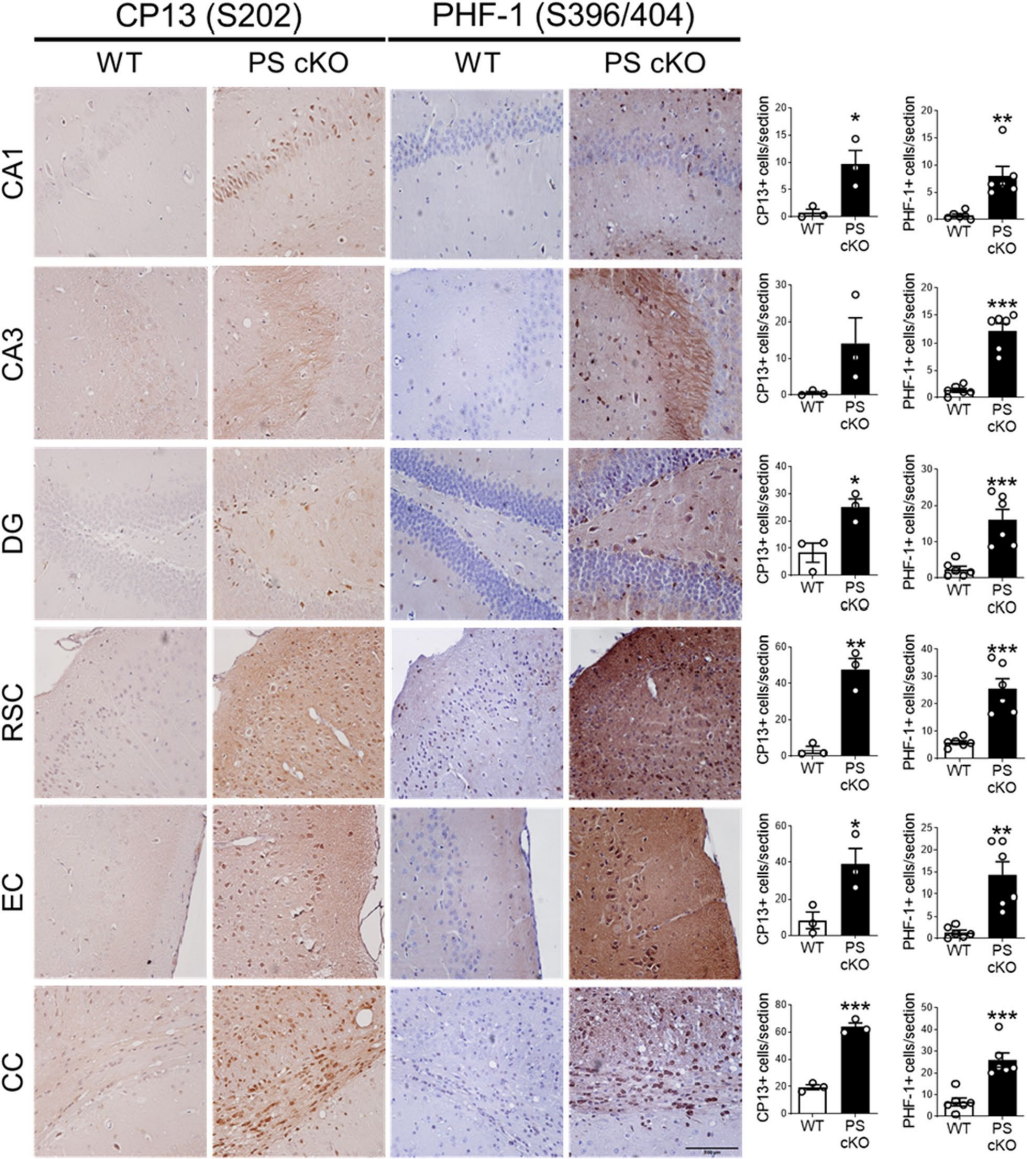
Absence of PS increases tau phosphorylation in memory-related brain regions. Left: Representative immunohistochemical images of phosphorylated Ser202 (CP13, left) and Ser396/404 (PHF-1, right) tau in 12 month-old control (WT) and PS cKO mice. Scale bar: 100 µm. Right: Quantitative analysis of total intensity and number of positive cells of CP13 (left) and PHF-1 (right) immunohistochemistry. Values represent mean ± s.e.m. (n = 3 slices/mice, 3 mice/group). Statistical analysis was determined by unpaired student’s *t*-test. *P < 0.05, **P < 0.01, ***P < 0.001. Abbreviations: CA1/CA3 hippocampus; CC, corpus callosum; DG, dentate gyrus; EC, entorhinal cortex; RSC, restrosplenial cortex

**Fig. 4:**
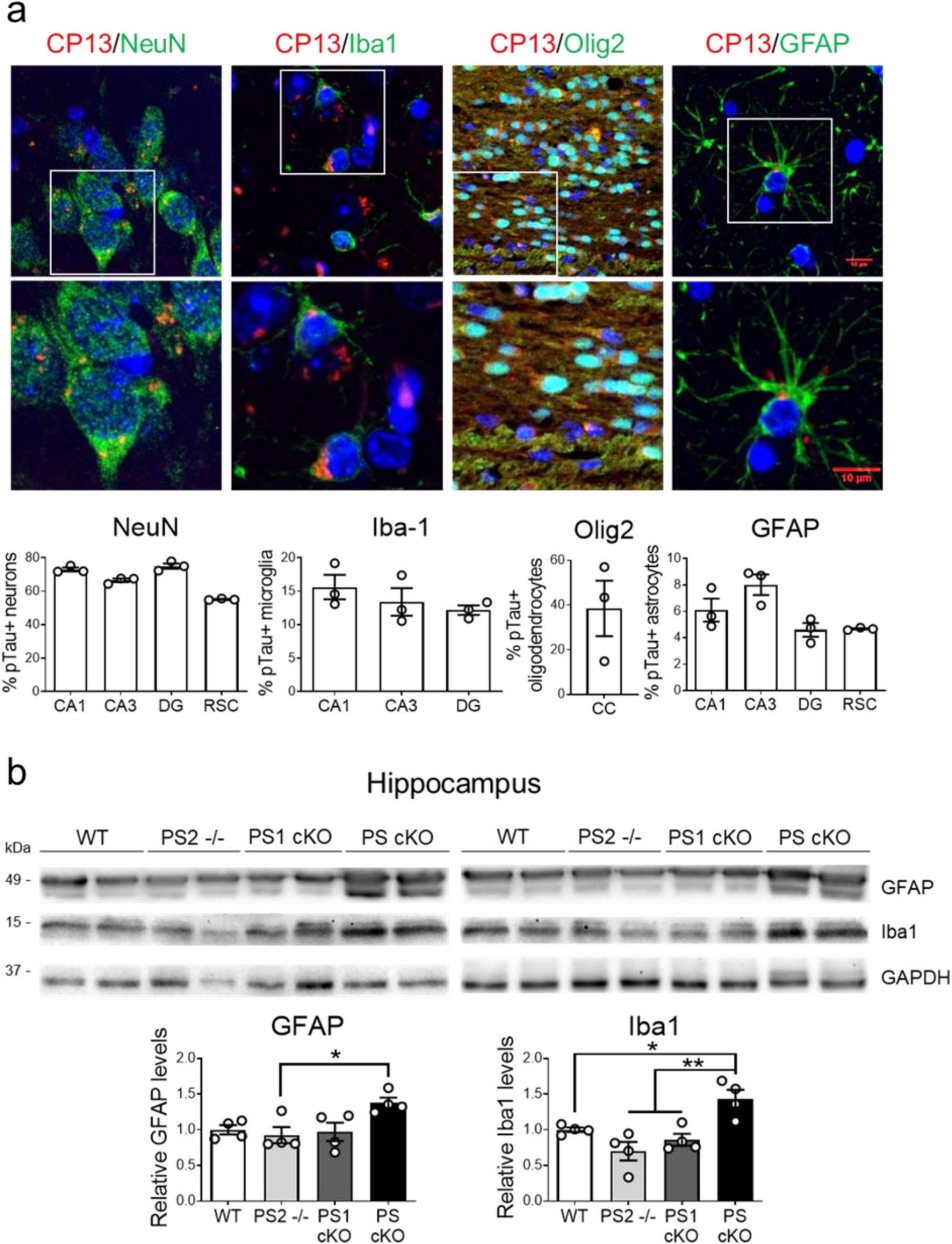
Inflammatory responses and accumulation of pathological tau in neurons and glial cells in PS cKO mice. **a** Representative immunohistological images and quantification (% of positive cells) showing accumulation of phosphorylated tau (CP13: Ser202, red) in neurons (NeuN, green), microglia (Iba1, green), oligodendrocytes (Olig2, green) and astrocytes (GFAP, green) of PS cKO mice at 12 months. Scale bar: 10 µm. Data represent percentage of pTau-positive cells ± s.e.m. (n = 3 mice/group, n = 5 sections/mouse). **b** Inflammatory responses in PS cKO mice. Western blot images (top) and quantitative analysis (bottom) of GFAP, Iba1 and GAPDH in control (WT), PS1 cKO, PS2^−/−^ and PS cKO mice at 9 months of age. Protein levels were normalized to GAPDH. Data represent relative fold levels ± s.e.m. (n = 4 mice/group). Statistical analysis was determined by one-way ANOVA followed by Tukey’s post hoc test. *P < 0.05, ** *P* < 0.001 compared to the indicated group

To investigate accurately the brain regions where tau pathology accumulates, we performed immunohistochemical analysis of phosphorylated tau using CP13 and PHF-1 antibodies in 12 month-old control and PS cKO mice. In the hippocampus, phosphorylated tau staining is present in neuronal projections and somatodendritic compartments of excitatory pyramidal neurons in PS cKO mice (Fig. 3). Quantitative analysis revealed significant increases of phosphorylated tau-staining and -positive neurons in the hippocampus (CA1, CA3, DG: dentate gyrus), retrosplenial (RSC) and entorhinal (EC) cortices, and corpus callosum (CC) of PS cKO mice (*t*-test, *P* < 0.05; Fig. 3, data not shown). Double immunostaining using CP13 and markers of neurons (NeuN), microglia (Iba1), oligodendrocytes (Olig2) and astrocytes (GFAP) revealed abundant phosphorylated tau in ~ 60–70% neurons, ~ 15% microglia and ~ 6–8% astrocytes in the hippocampus and restrosplenial cortex of PS cKO mice (Fig. 4a). Interestingly, phosphorylated tau is highly abundant in oligodendrocytes (40%) in the CC of PS cKO mice (Fig. 4a). CP13 immunoreactivity was very low or absent for reliable quantification of phosphorylated tau in distinct cell types in control mice (Additional file 1: Fig. S1).

To study whether increased tau phosphorylation in glial cells was accompanied by inflammatory responses, we next examined astrocytic (GFAP) and microglial (Iba1) markers in the hippocampus of WT, PS2^−/−^, PS1 cKO and PS cKO mice at 9 months of age (Fig. 4b). Quantitative and statistical analysis revealed a genotype effect in GFAP (*P* < 0.05) and Iba1 (*P* < 0.01) levels indicating differences among groups. Post-hoc analysis revealed significant enhanced GFAP and Iba1 only in PS cKO mice (*P* < 0.05). These results demonstrate that phosphorylated tau is present in neurons and glial cells of PS cKO mice during inflammation and neurodegeneration.

### Partial loss of PS/γ-secretase function enhances phosphorylation and aggregation of human tau

**Fig. 5:**
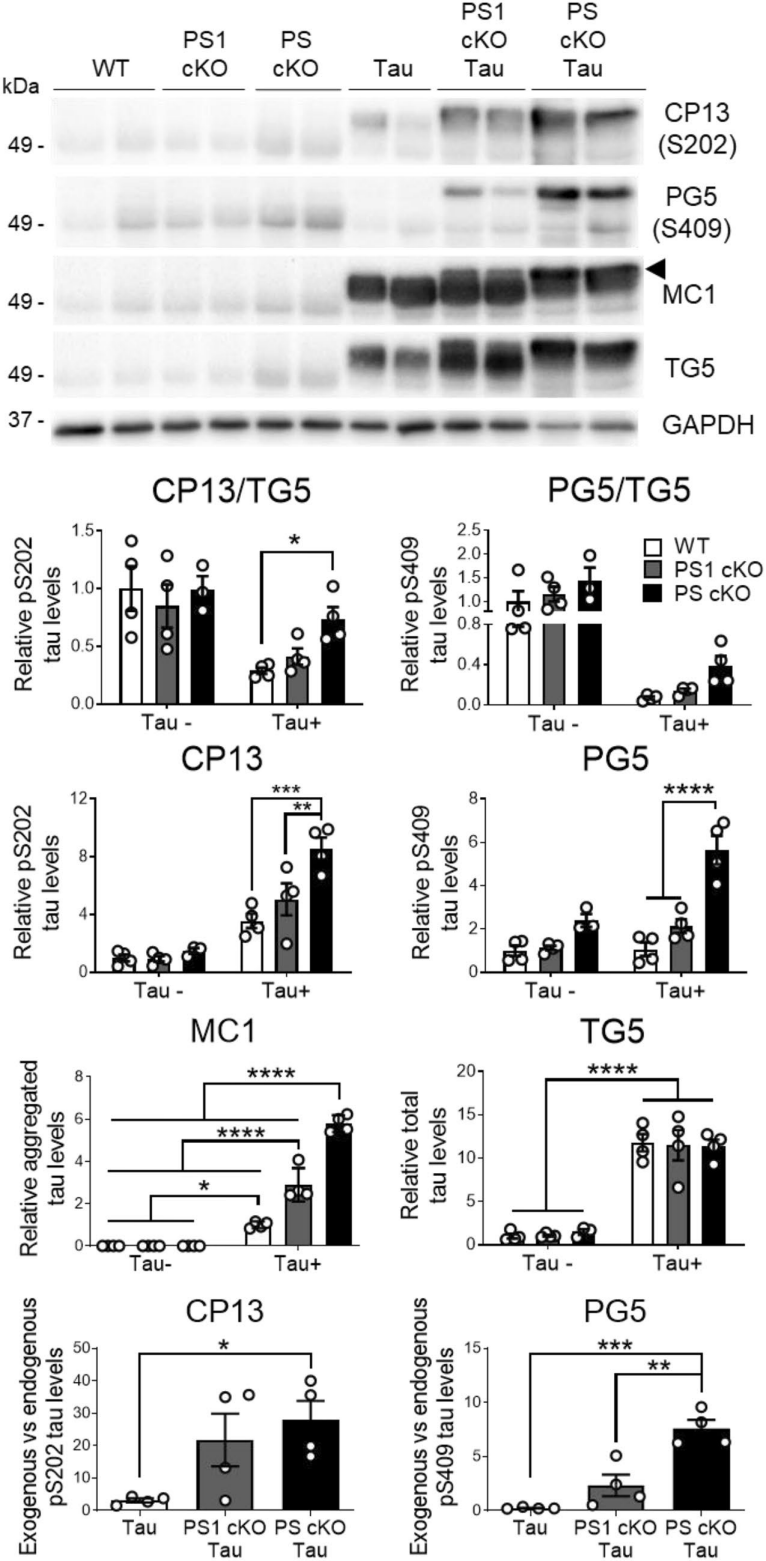
Lack of PS increases human tau phosphorylation and aggregation in the hippocampus. Biochemical analysis of phosphorylated tau in the hippocampus of WT, PS1 cKO, PS cKO, Tau, PS1 cKO;Tau and PS cKO;Tau mice at 6 months of age. Western blotting was performed using antibodies against human tau (TG5), phosphorylated tau (CP13: Ser202; PG5: Ser409), and aggregated tau (MC1). TG5 was incubated in the same membrane of CP13 blotting after stripping. The arrowhead indicates the quantified MC1-labeled high molecular weight aggregated tau species. Quantification of exogenous (human) vs endogenous (murine) phosphorylated tau levels are shown at the bottom. Phosphorylated and total tau levels are normalized to GAPDH. Values represent mean ± s.e.m. (n = 4 mice/group). Statistical analysis was determined by one-way ANOVA followed by Tukey’s post hoc tests. *P < 0.05, ** *P* < 0.001, *** *P* < 0.0001

We next investigated the role of PS in human tau pathology in novel PS1 cKO;Tau **(**PS1 f/f;CamKIIα-Cre;Tau) and PS cKO;Tau **(**PS1 f/f;PS2^−/−^;CamKIIα-Cre;Tau) mice obtained by crossing PS1 cKO and PS cKO mice with mutant Tau transgenic (PS19) mice expressing the FTD-linked P301S mutation in excitatory neurons [73]. Soluble plus aggregated tau species were extracted from hippocampus as reported [51]. Compared to non-transgenic mice, total tau (TG5) was similarly increased (~ 8–12 fold) in all tau transgenic groups independently of PS expression (Fig. 5). Interestingly, we noticed a decrease of GAPDH, which was used for normalization, in PS cKO;Tau mice, as previously observed in AD brain [10]. Phosphorylated tau at the Cdk5/GSK3β residue Ser202 (CP13) was increased in the hippocampus of 6 month-old PS1 cKO;Tau and/or PS cKO*;*Tau mice compared to Tau mice (Two-way ANOVA, CP13: tau genotype effect, *P* < 0.0001; PS effect, *P* < 0.01; tau x PS interaction effect, *P* < 0.01) (Fig. 5). In addition, the PG5 antibody that recognizes the PKA/AMPK-dependent tau phosphorylation at Ser409 present in PHFs [34], detected a prominent low-migrating band significantly increased in PS cKO;Tau hippocampus (tau effect, *P* < 0.001; PS effect, *P* < 0.0001; tau x PS interaction effect, *P* < 0.01; Fig. 5). Notably, the ratio of exogenous (human)/endogenous (murine) phosphorylated tau was increased in PS1 cKO and/or PS cKO hippocampi (Fig. 5 bottom).

**Fig. 6:**
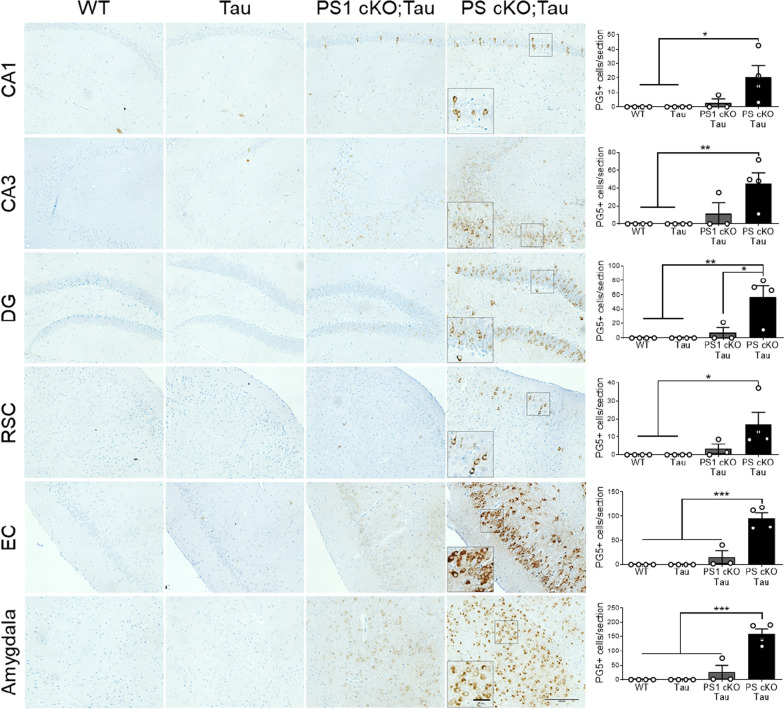
Increased pathological PKA-mediated tau phosphorylation in Tau mice lacking PS. Left: Representative immunohistochemical images of phosphorylated tau (PG5: pSer409) in 6 month-old non- and tau transgenic mice. Inset: magnified images showing somatic PG5 staining in PS cKO;Tau brains. Scale bar: 100 μm. Right: Quantitative analysis of number of PG5-positive cells/section in the indicated brain region in littermate control (WT), Tau, PS1 cKO;Tau and PS cKO;Tau mice. Values represent mean PG5-positive cells ± s.e.m. (3 slices per mouse, 4–5 mice/group). Statistical analysis was determined by two-way ANOVA followed by Tukey’s post hoc tests. *P < 0.05, **P < 0.01, ***P < 0.001. Abbreviations: Amygdala: basolateral amygdala; CA1/CA3 hippocampus; DG, dentate gyrus; EC, entorhinal cortex; RSC, retrosplenial cortex

Immunohistochemical analysis revealed enhanced PG5 staining in neuronal soma and axons of PS cKO;Tau mice at 6 months, and sporadically of PS1 cKO;Tau mice (Fig. 6). In PS cKO;Tau mice, PG5-positive cells were significantly increased in glutamatergic pyramidal neurons of CA1, CA3 and DG hippocampus (genotype effect, *P* < 0.05), restrosplenial cortex (RSC: genotype effect, *P* < 0.05), entorhinal cortex (EC: genotype effect, *P* < 0.0001) and basolateral amygdala (genotype effect, *P* < 0.0001) (Fig. 6). Compared to PS1 cKO;Tau mice, the average of PG5-positive cells in CA1/CA3 hippocampus and RSC of PS cKO;Tau mice were enhanced but not significantly (*P* > 0.05; Fig. 6).

**Fig. 7:**
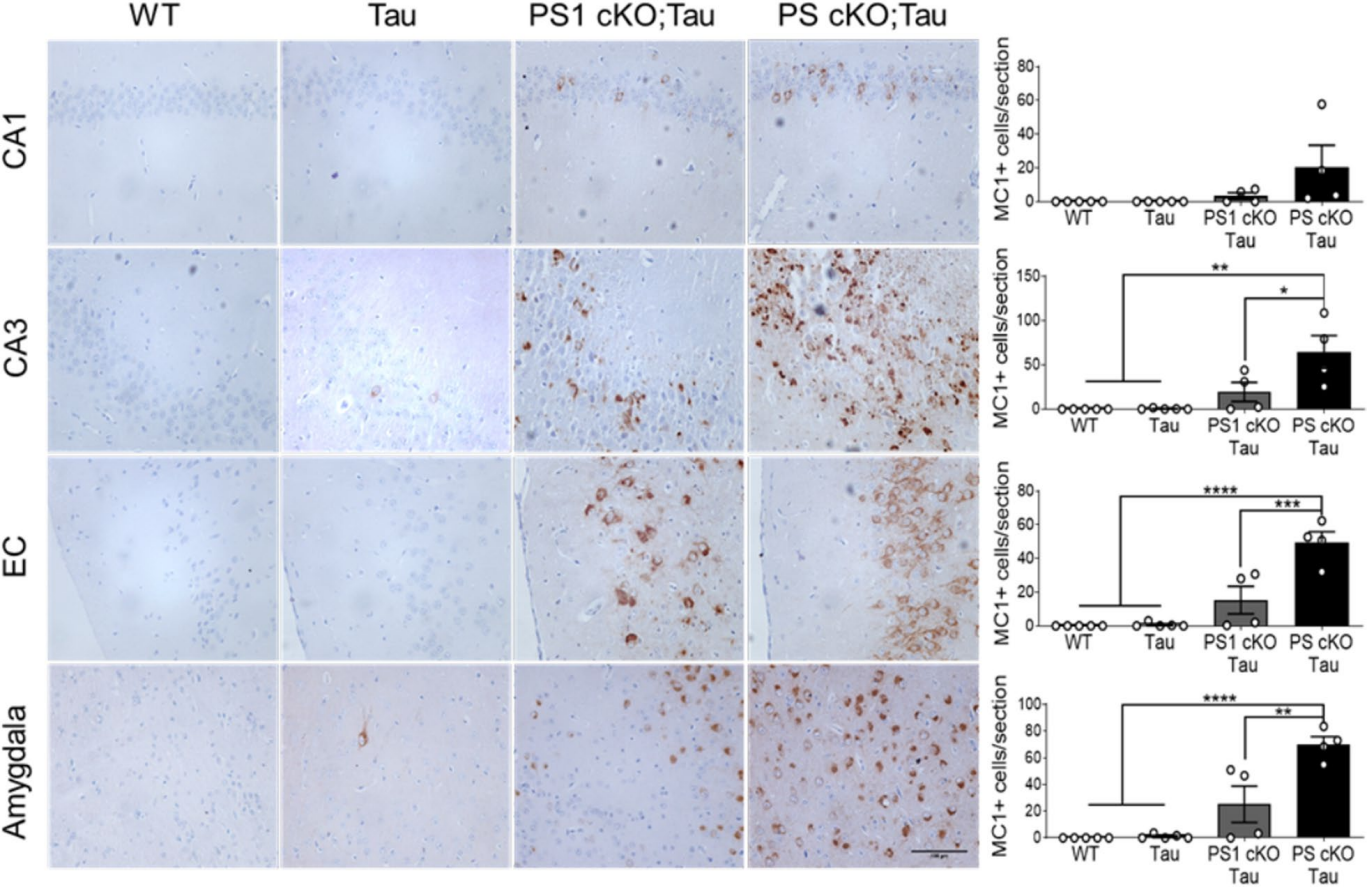
MC1 staining reveals aggregated human tau in Tau mice lacking PS1 and both PS. Immunohistochemical images (left) and quantitative analysis (right) of MC1 stained sections in 6 month-old control (WT), Tau, PS1 cKO;Tau and PS cKO;Tau mice. Scale bar: 100 μm. Values represent mean of MC1-positive cells/section ± s.e.m. (n = 3 slices per mouse, 4 mice/group). Statistical analysis was determined by one-way ANOVA followed by Tukey’s post hoc tests. *P < 0.05, **P < 0.01, ***P < 0.001, ****P < 0.0001. Abbreviations: Amygdala: basolateral amygdala; CA1/CA3 hippocampus; EC, entorhinal cortex ,

To investigate the role of PS on tau aggregation, we next used the MC1 antibody recognizing conformational pathological tau (aa 312–322) in AD brain (Table 1). Biochemical analysis of cortical and hippocampal lysates showed a significant increase of a retarded aggregated tau band in PS1 cKO;Tau and PS cKO;Tau mice compared to Tau mice (genotype effect, *P* < 0.0001; Fig. 5 and data not shown)). Indeed, there were significant differences in MC1 between PS1 cKO;Tau and PS cKO;Tau mice (*P* < 0.0001). Immunostaining with MC1 antibody showed neuronal somatodendritic aggregated tau staining in different brain regions, including the hippocampus, EC and basolateral amygdala in 6 month-old PS1 cKO;Tau and PS cKO;Tau mice (Fig. 7). Quantification and statistical analysis revealed genotype differences in MC1-positive cells in CA3 (*P* < 0.001), EC (*P* < 0.0001) and basolateral amygdala (*P* < 0.0001) (Fig. 7), indicating significant changes of aggregated tau in PS-deficient tau mice. Compared with PS1 cKO;Tau mice, MC1-positive cells were significantly elevated in PS cKO;Tau mice in the hippocampus (CA3: *P* < 0.05), EC (*P* < 0.001) and amygdala (*P* < 0.01) (Fig. 7). These results indicate that partial loss of PS function results in human tau phosphorylation and aggregation.

### PS regulates neurofilament cytoskeleton structures

**Fig. 8:**
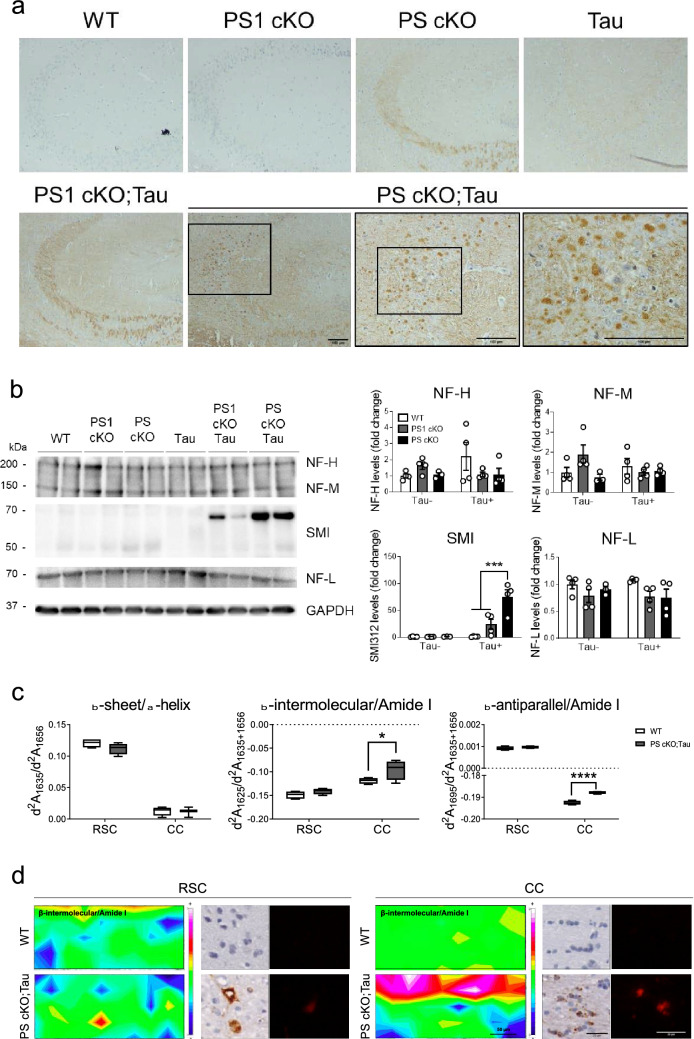
PS deficiency causes abnormal NF levels and pathological β-sheet protein structures. **a** Representative immunohistological images of NF-L in the hippocampus of 6 month-old WT, PS1 cKO, PS cKO, Tau, PS1 cKO;Tau and PS cKO;Tau mice. Insets: magnified images indicated by squares showing prominent NF-L somatic staining in hippocampal neurons of PS cKO;Tau mice. Scale bar = 100 μm. **b** Left: Biochemical analysis of hippocampal lysates using SMI312 (NF-M/H) and NF-L antibodies. Right: Quantification of NF-H (~ 200 kDa), NF-M (~ 150 kDa), SMI-labeled (~ 60–70 kDa) and NF-L (~ 70 kDa) bands in independent membranes. Protein levels were normalized to GAPDH. Values represent mean ± s.e.m. (n = 4 mice/group). Statistical analysis was determined by two-way ANOVA followed by Tukey’s post hoc tests. ***P < 0.001. **c** Synchrotron-based µFTIR analysis of the retrosplenial cortex (RSC) and corpus callosum (CC) of WT and PS cKO;Tau mice at 6 months of age. Second derivative absorbances (d^2^A) of β-sheet/α-helix (d^2^A_1635_/d^2^A_1656_), and β-intermolecular/Amide I (d^2^A_1625_/d^2^A_1635+1656_) and β-antiparallel/Amide I (d^2^A_1695_/d^2^A_1635+1656_) protein structures. Values represent the minimum, the maximum and the median of the average of 100 spectra/mouse (n = 4 mice/group). Statistical analysis was determined by one-way ANOVA followed by Sidak’s post hoc tests. *P < 0.05, ***P < 0.001, ****P < 0.0001. **d **Representative infrared heat maps of β-intermolecular/Amide I (left images; scale bar = 50 μm) and consecutive immunohistological sections of aggregated tau  detected by MC1 staining (middle images) and Congo red staining (right images; scale bar = 25 μm) in the RSC and CC of WT and PS cKO;Tau mice.

PS1 regulates the assembly of neurofilaments (NF) and *PSEN1* mutations elevate levels of NF light chain (NF-L) in cerebral spinal fluid (CSF) of AD patients [19, 71]. To examine whether neuronal PS regulate NF dynamics in the adult brain, we next performed immunohistochemical and biochemical analysis using antibodies against NF-L and SMI312, an antibody specific for phosphorylated NF medium (NF-M) and high (NF-H) chains. In hippocampus and basolateral amygdala, NF-L and SMI312 stainings were enhanced in neuronal projections and/or soma of 6 month-old PS cKO and PS1 cKO;Tau or PS cKO;Tau mice, respectively, a pattern not observed in WT, PS1 cKO and Tau mice (Fig. 8a; Additional file 1: Fig. S2). Notably, biochemical analysis revealed global decreased NF-L and NF-H levels in the hippocampus of PS1 cKO;Tau and PS cKO;Tau mice (PS effect, *P* < 0.05; Fig. 8b). Interestingly, we found a ≈70 kDa band increased in the hippocampus of PS cKO;Tau mice likely related to SMI312-stained aggregated forms (tau effect, *P* < 0.0001; PS effect, *P* < 0.001; tau x PS interaction effect,*P* < 0.001;Fig. 8b).These results indicate abnormal neurofilament-related protein structures in Tau mice lacking neuronal PS.

### Synchrotron infrared microspectroscopy reveals protein β-sheet aggregates in PS cKO;Tau brains

To analyze in depth protein structural and aggregation changes directly in brain tissue of PS-deficient Tau mice, we performed synchrotron-based μFTIR, a sensitive label-free and non-destructive in situ imaging technique previously used to characterize amyloid molecular structures in AD [3, 53]. We evaluated chemical composition and molecular structures in RSC and CC of confirmed pathological MC1- and Congo red-stained positive consecutive brain sections of 6 month-old WT and PS cKO;Tau mice (Fig. 8c, d). As MC1, Congo red staining was absent in WT mice and low in Tau mice at this age (not shown). Synchrotron infrared radiation showed clear differential biochemical profiles of the two analyzed brain regions in lipid oxidation (C = O/CH_2_ and C = CH/CH_2_) and protein/lipid ratio (Amide I/CH_2_), but without significant changes among genotypes (Additional file 1: Fig. S2, ). To extend the histochemical and biochemical results showing conformational and aggregated tau in PS-deficient Tau mice, protein β-sheet conformations were studied after second derivation of the infrared spectra. Specifically, we measured intramolecular (d^2^A_1635_/d^2^A_1656_), intermolecular (d^2^A_1625_/d^2^A_1635+1656_) and antiparallel (d^2^A_1695_/d^2^A_1635+1656_) protein structures corresponding to non-aggregated, aggregated and oligomeric β-sheet structures, respectively [2, 5, 8]. While the relative amount of intramolecular β-sheet structures was similar in both genotypes, the intermolecular (genotype effect, *P* < 0.05; area effect, *P* < 0.0001) and antiparallel (genotype effect, *P* < 0.0001; area effect, *P* < 0.0001; genotype x area interaction effect, *P* < 0.0001) β-sheet structures were significantly higher in the CC of PS cKO;Tau mice (Fig. 8c, d). This result, together with Congo red staining, indicates abnormal levels of aggregated oligomeric and fibrillar β-sheet protein conformations in the CC of PS cKO;Tau mice.

### PS-dependent effects in hippocampal-dependent memory

**Fig. 9:**
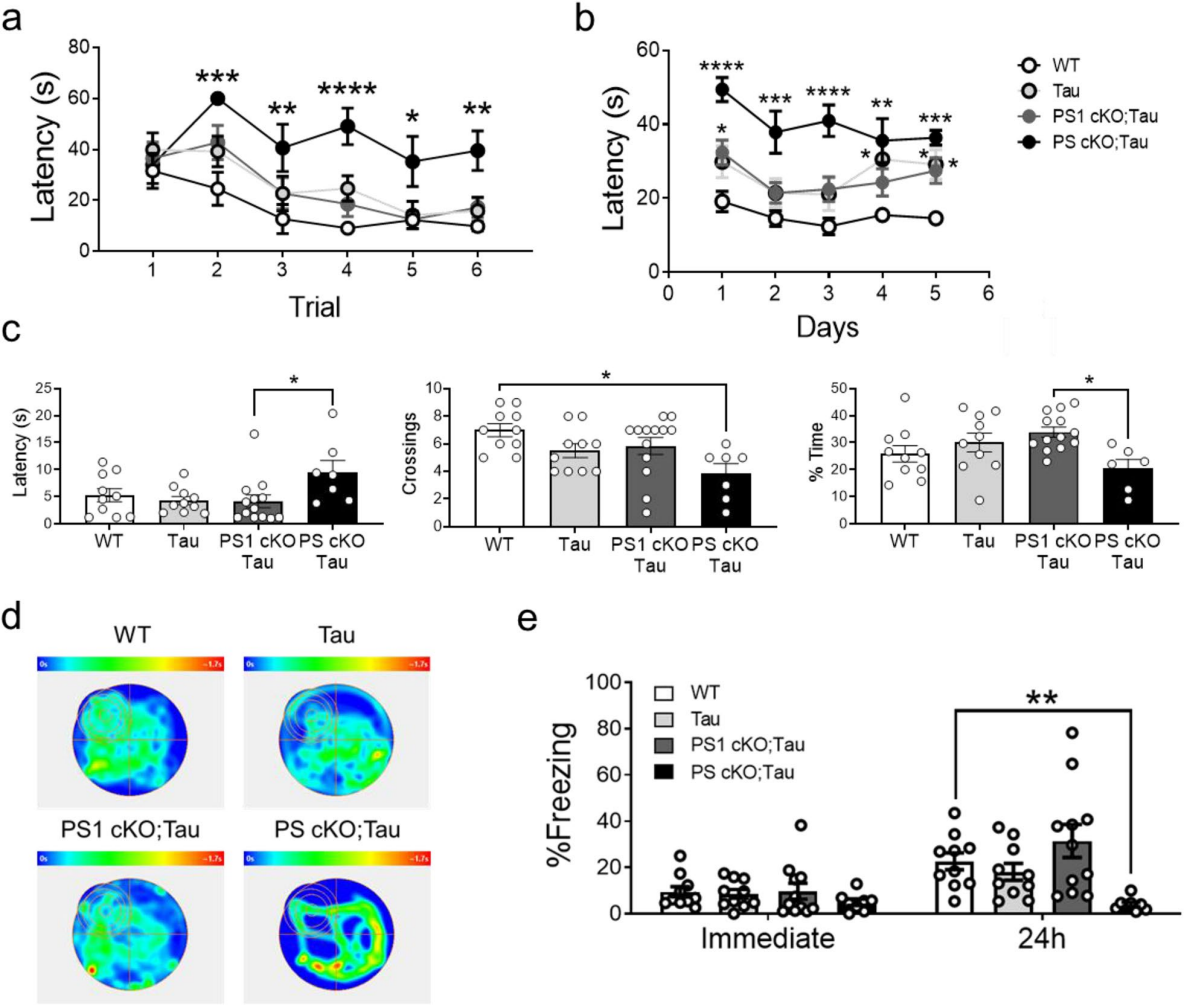
Loss of PS function causes hippocampal-dependent memory deficits in Tau transgenic mice. Morris water maze and CFC was performed with 6 month-old WT, Tau, PS1 cKO;Tau and PS cKO;Tau mice. **a** Visible platform phase consisted in six trials and platform latency in seconds was measured. **b** During learning phase, which consisted in six trials during five consecutive days, latency to the platform in seconds was measured. **c** Latency to the first entry to the target quadrant in seconds, number of crossings to the target quadrant and percentage of time in the target quadrant in the first 30 s of the test were analyzed. **d** Representative heat map of each analyzed group during the probe test. **e** CFC showing the percentage of freezing time immediately and 24 h after conditioning. Values represent mean % freezing time ± s.e.m. (n = 7–10 mice/group). Statistical analysis was determined by one or two-way ANOVA followed by Tukey’s post hoc tests. *P < 0.05, **P < 0.01, ***P < 0.001, ****P < 0.0001

To analyze the effects of tau pathology on hippocampal-dependent memory, we next tested 6 month-old littermate control, Tau, PS1 cKO;Tau and PS cKO;Tau mice in the Morris water maze (MWM) and contextual fear conditioning (CFC) tasks. Swimming speed during training in the visible platform test were similar among all groups (*P* > 0.05), ruling out the possibility of motor and visual deficits. Although no differences were detected in the first trial, there were significant latency changes depending on genotype and trial (*P* < 0.0001; Fig. 9a). Indeed, latencies of PS cKO;Tau mice were significantly higher in trials 2–6 likely reflecting lack of motivation (Fig. 9a). During training in the hidden platform task, mice performed differentially depending on genotype and day (Two-way ANOVA: genotype effect, *P* < 0.0001; day effect, *P* < 0.01). Tau, PS1 cKO;Tau and PS cKO;Tau mice exhibited significantly longer latencies that control mice (*P* < 0.0001; Fig. 9b), whereas PS cKO;Tau mice spent significant longer latencies than Tau and PS1 cKO;Tau mice (Two-way ANOVA: *P* < 0.0001). In the probe trial on day 6, PS cKO;Tau mice spent more time to find the target quadrant, and crossed less often and spent less time in the target quadrant than the rest of groups (one-way ANOVA: latency, crossing and occupancy in target quadrant,* P* < 0.05; Fig.9,cd). In contextual fear conditioning, freezing responses immediately after shock were similar in all experimental groups, suggesting no differences in sensing the footshock (Fig. 9e). By contrast, freezing responses at 24 h were reduced in PS cKO;Tau mice compared to the rest of groups. Indeed, two-way ANOVA showed main effects of genotype (*P* < 0.01) and time (*P* < 0.001) (Fig. 9e). Post-hoc analysis revealed only significant differences in freezing percentage between control and PS cKO;Tau mice (*P* < 0.01). These results indicate PS dosage-dependent effects on hippocampal-dependent spatial and associative memory in Tau transgenic mice.

### Accumulation of synaptic tau and reduced synaptic proteins in Tau mice

**Fig. 10:**
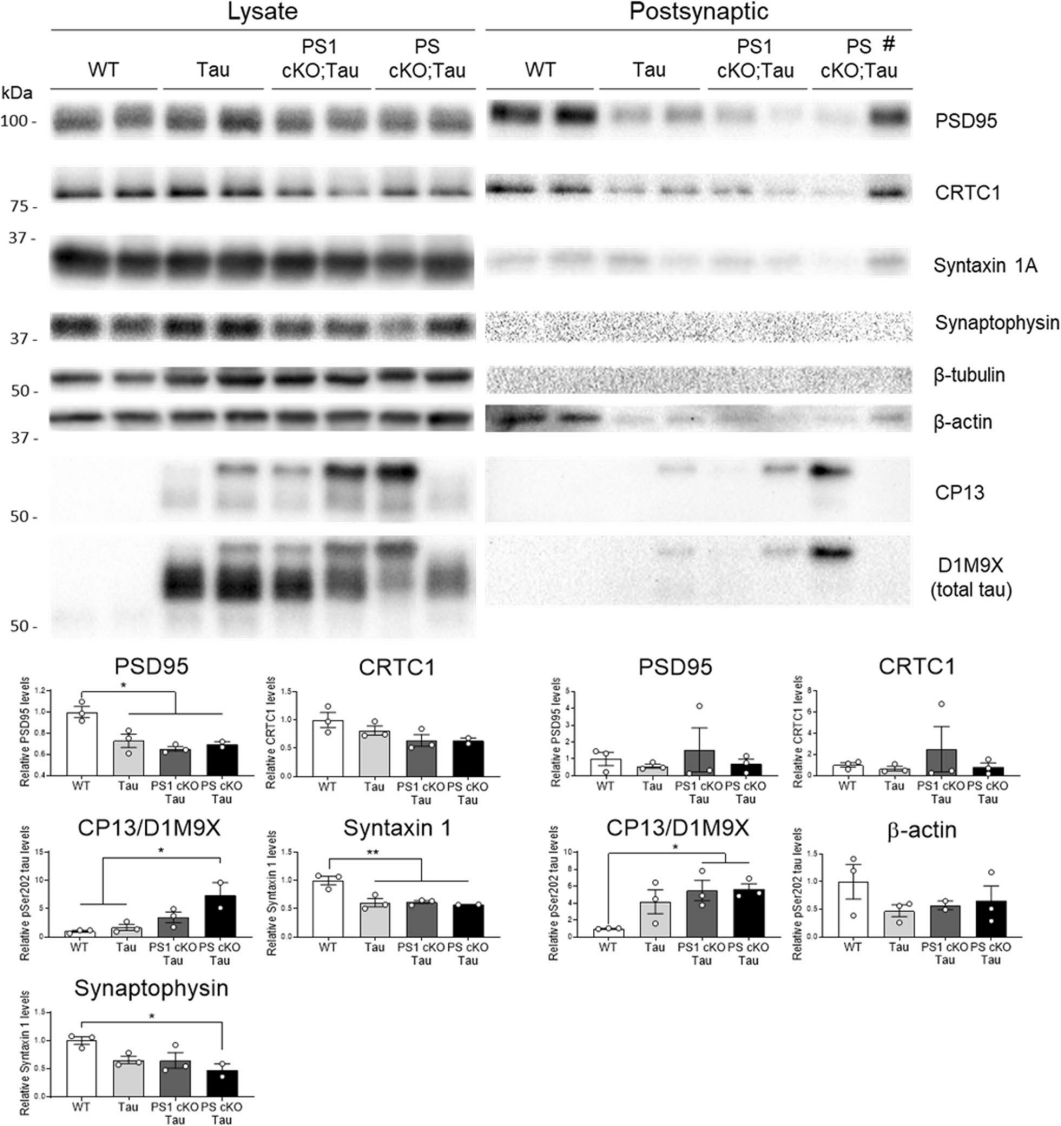
Reduced synaptic proteins and accumulation of synaptic tau in PS-deficient Tau mice. Western blot images (top) and quantification (bottom) of synaptic proteins and phosphorylated Ser202 (CP13) and total (D1M9X) tau in hippocampal lysates (left) and purified postsynaptic fractions (right) of WT, Tau, PS1 cKO;Tau and PS cKO;Tau mice at 6 months. # indicates a young 3 month-old PS cKO;Tau mouse. Protein levels were normalized to β-tubulin in the lysates and total loaded protein (1 μg) in postsynaptic fractions. D1M9X antibody was used for reblotting after CP13 membrane stripping. Notice the lack of typical presynaptic proteins (syntaxin 1A, synaptophysin, β-tubulin) in the postsynaptic fractions. Values represent mean fold ± s.e.m. (n = 3 mice/group). Statistical analysis was determined by one-way ANOVA followed by Tukey’s post hoc tests. *P < 0.05

Abnormal accumulation of tau at synapses induces synapse dysfunction, instability and loss [29, 33, 73]. To investigate the synaptic effects of tau pathology we next examined synaptic proteins in purified synaptosomal and pre- and post-synaptic fractions of hippocampus of 6–9 month-old WT and Tau mice. Biochemical analysis of synaptic fractions revealed the presence of phosphorylated tau (PHF-1; *t*-test: synaptosome, *P* < 0.05; presynaptic, *P* < 0.05; postsynaptic *P* < 0.05), and decreased PSD95 and CRTC1 (synaptosome, *P* < 0.05; postsynaptic, *P* < 0.05) and synaptophysin (presynaptic, *P* < 0.05) in synaptic compartments of Tau transgenic mice (Additional file 1: Fig. S3). Finally, we examined synaptic proteins in hippocampal lysates and postsynaptic fractions of WT, Tau, PS1 cKO;Tau and PS cKO;Tau mice at 6 months of age. Interestingly, PSD95, CRTC1, syntaxin 1A and synaptophysin (One-way ANOVA: *P* < 0.05) were decreased in the hippocampus of PS cKO;Tau mice coinciding with enhanced phosphorylated/total tau levels (One-way ANOVA: *P* < 0.05; Fig. 10). In postsynaptic fractions obtained from purified synaptosomes, PSD95, CRTC1 and β-actin were reduced coinciding with higher synaptic phosphorylated/total tau in mice expressing tau (One-way ANOVA: *P* < 0.05; Fig. 10). Classical presynaptic proteins, including syntaxin 1A, synaptophysin and β-tubulin, were excluded from the postsynaptic fractions confirming the validity of our purification approach (Fig. 10; Additional file 1: Fig. S3). By contrast, PSD95 and CRTC1 were unchanged in postsynaptic fraction of a 3 month-old PS cKO;Tau mouse lacking synaptic tau (indicated as # in Fig. 10). These results indicate that synaptic pathological tau is associated with altered synaptic proteins in the hippocampus of Tau mice.

## Discussion

Inclusions of aggregated hyperphosphorylated tau are pathological hallmarks of sporadic and *Tau-* and *PS1/PSEN1*-linked autosomal dominant neurodegenerative tauopathies [4, 16, 54]. To develop novel therapeutic interventions in these memory disorders, it is critical to elucidate the cellular mechanisms linking PS/γ-secretase and tau pathology. However, characterization of such mechanisms in vivo is challenging because requires precise mouse models modeling tau aggregation. This study shows that PS/γ-secretase regulates tau homeostasis in excitatory neurons by tightly controlling tau phosphorylation and aggregation. Accordingly, partial loss of neuronal PS function in mice results in age-dependent tau phosphorylation and aggregation in excitatory neurons of AD-related vulnerable memory regions. Accumulation of pathological tau occurs at synapses and also in glial cells and is associated with inflammation, neurodegeneration and memory deficits. We conclude that disruption of PS/γ-secretase compromises tau homeostasis and recapitulates key pathological features of tauopathies.

Neurodegenerative tauopathies linked to inherited *PSEN1* mutations are characterized by exacerbated cerebral tau pathology, including NFTs and NFT-associated neurites [25, 30, 37, 61, 63, 71]. Our findings showing that PS/γ-secretase deficiency in glutamatergic neurons results in age- and gene dosage-dependent tau phosphorylation and aggregation raises the possibility that loss of PS function mediates tau pathology in dementia. Although total loss of PS function enhances endogenous or human tau phosphorylation [46, 57], our results go a step further showing that partial PS deficiency potentiates tau aggregation, which may include β-sheet oligomeric structures. However, in contrast to typical studies on protein aggregates using biochemical methods in brain that alter protein conformation and states, our in situ histochemical and infrared spectroscopy imaging analyses are sensitive and non-invasive techniques. On the other hand, previous studies showed that familial AD-linked *PS1* mutants may act via a dominant-negative effect on APP processing [36, 64]. Particularly, the *PSEN1* L435F mutation decreases total Aβ while enhancing Aβ42/Aβ40, amyloid deposition and tau phosphorylation causing synaptic deficits and neurodegeneration [72]. Consistent with a role of PS on tau pathology, mutant PS1 increases GSK3β-mediated tau phosphorylation [17, 65, 72], and loss of neuronal PS increases tau phosphorylation at Cdk5/GSK3β sites through elevation of p25 and Cdk5 activity (data not shown; [57]). It is interesting that PS/γ-secretase deficiency elevates endogenous APP CTFs and decreases Aβ [74], which raises the possibility that mutant PS1 could accelerate tau pathology through altered APP processing, Aβ changes and/or other cellular effectors. In addition, loss of PS enhances PKA/AMPK-dependent tau phosphorylation at Ser409, a site characteristic of PHFs in AD brain [34]. This is supported by a recent study indicating that PS1 negatively affects cAMP/PKA signaling during neurite outgrowth [18], whereas PKA/JNK mediates Aβ-induced tau pathology in PS1/APP mice [69]. Alternatively, tau phosphorylation and tau-mediated impaired cognition was recently linked to age-related calcium dysregulation in non-human primates [11]. Since PS regulate cellular pathways affecting calcium homeostasis [14], it is plausible that loss of PS function could induce PKA-mediated tau phosphorylation by exacerbating age-related calcium disturbance. These results raise the possibility that some familial AD-linked PS mutations may enhance tau pathology via a partial loss of function mechanism.

Pathological studies show that tau accumulates progressively and preferentially in excitatory neurons of limbic regions in AD brain [7, 21, 31, 38, 68]. Notably, tau pathology occurs prominently in neurons of memory-related AD vulnerable brain regions in PS-deficient Tau transgenic mice. Considering that human tau expression in the EC causes tau propagation, synaptic and excitatory neuron loss, and memory deficits [12, 22], it is plausible that loss of PS in excitatory neurons could drive tau spreading to interconnected neuronal circuits and/or cell types (see below). Similar to mutant *PSEN1* cases [50, 71], tau accumulation in PS/γ-secretase-deficient mice is associated with brain degeneration, neuronal loss and abnormal NF cytoskeletal alterations, pointing to a causal relationship between these events. In support of this, PS and tau convergence on impairing axonal transport through brain-derived neurotrophic factor [46]. Importantly, pre-symptomatic familial AD-linked PS1 carriers show elevated NF-L in CSF/serum that predicts the onset and correlates with progression of clinical symptoms [52, 56]. Despite the clear link of pathological tau and NF-L in biological fluids of *PS* mutant carriers, the relationship of PS, tau and neurofilament is still unclear. It is known that PS1 regulates NF assembly, whereas familial AD-linked *PSEN1* mutations cause cytoskeletal abnormalities associated with increased tau phosphorylation, release from microtubules and binding to NF [19, 50, 71]. Considering that NF-L is a established marker of neurodegeneration, changes in total/phosphorylated NF could reflect the neurodegenerative process occurring in PS cKO;Tau mice.

Compelling evidence indicates that synaptic tau is a key factor contributing to synapse pathology in AD [32, 49]. Of relevance, the presence of synaptic tau and reduced presynaptic and postsynaptic proteins in the hippocampus coincide with exacerbated hippocampal-dependent memory deficits in PS-deficient Tau mice. Among the altered synaptic proteins, the synaptonuclear factor CRTC1 was recently linked to memory loss at early AD pathological stages [45], suggesting that altered synaptic CRTC1 may be involved in synapse pathology in tauopathies. Our observation also agrees with presynaptic and postsynaptic plasticity impairments and synapse loss occurring in Tau P301S mice during aging [15, 73], and the fact that synaptic tau induces synapse dysfunction and loss [29, 33, 73]. Although Aβ and tau synergize on synaptic dysfunction [9], our results indicate that abnormal synaptic tau could mediate itself synapse pathology. Together, these results suggest a close relationship between synaptic tau, synapse pathology and behavioral symptoms. Additional studies will be necessary to discern the underlying PS-dependent mechanisms of synaptic tau accumulation and its contribution to memory loss.

Pathological tau is present in glial cells and is accompanied by elevated inflammatory markers in *PS* mutant mice suggesting a link between inflammation and tau pathology. Phosphorylated tau accumulates in neurons, microglia, astrocytes and oligodendrocytes in PS cKO brain, an intriguing result considering that PS1 is selectively inactivated in excitatory neurons [57]. It is possible that released tau in *PS*-deficient neurons could lead to its uptake by glial cells or be the result of microglial phagocytosis of degenerating neurons containing tau. Indeed, tau aggregates are present in astrocytes and microglia in aged and degenerated brains [35, 47]. Of relevance, internalization of extracellular tau by microglia and astrocytes is mediated by fractalkine receptor and Transcription Factor EB, respectively [6, 41] , and astrocytic complement C3 and its microglial receptor C3aR mediates neuronal tau pathology in AD brain and PS19 transgenic mice [39]. The pathological consequences of astrocytic tau accumulation are not clear but, as suggested in transgenic mice, they may contribute to glial degeneration, tau spreading and synapse dysfunction [28, 41, 48]. In contrast to tau accumulation in neurons in AD brain, tau is also aggregated in astrocytes and oligodendrocytes in CBD and PSP [35]. The presence of pathological tau in oligodendrocytes may be critical in tauopathies, as suggested by oligodendrocyte loss caused by tau propagation in oligodendrocytes [44]. In this regard, synchrotron infrared microspectroscopy detected intermolecular and antiparallel β-sheets in the CC of PS cKO;Tau mice. The peak of intermolecular β-sheet structures corresponds to a spectral signature for disease-associated oligomeric and/or fibrillar protein aggregates, including Aβ and tau [2, 5], whereas the antiparallel β-sheet peak is linked to oligomeric non-fibrillar Aβ aggregates [8]. Although the contribution of pathological tau to this spectroscopic signature in vivo is still poorly understood, the presence of β-sheet protein structures together with Congo red staining in these mice lacking Aβ pathology suggests that they could correspond to oligomeric and fibrillar tau aggregates. Importantly, whereas the specific protein composition of Aβ aggregates and tau filaments is mainly attributed to intermolecular β-sheet structures [2, 8, 53], in the present study we measured the chemical composition along the brain tissue, which allowed detection of unaltered intramolecular β-sheet structures as revealed by unchanged β-sheet/α-helix ratio. To the best of our knowledge, this is the first time that infrared microspectroscopy reveals differences in pathological protein aggregation between control and amyloid plaque-free transgenic AD mice.

In summary, PS/γ-secretase regulates tau homeostasis and its partial loss leads to progressive tau pathology, memory deficits and neurodegeneration. Future studies should discern the mechanisms leading to tau aggregation and the functional consequences of neuronal, glial and synaptic tau accumulation in familial AD.

## Supplementary Information

**Additional file 1**. CP13 immunostaining in control mice. CP13 staining (red) is barely detected in neurons (NeuN; green), microglia (Iba1; green), oligodendrocytes (Olig2; green) and astrocytes (GFAP; green) in brain sections of control (WT) mice. Insets: magnified images of the indicated selected regions of CA3, CA1, CC and DG (upper images) are shown at the bottom. Abbreviations: CA1/CA3 hippocampus; DG: dentate gyrus; CC, corpus callosum.

**Additional file 2**. NF staining and synchrotron-based µFTIR analysis of PS-deficient Tau mice. **A**, Immunohistological images of SMI312 (NF-H/M) in the basolateral amygdala of WT, PS1 cKO, PS cKO, Tau, PS1 cKO;Tau and PS cKO;Tau mice at 6 months of age. Insets: magnified images of the indicated left square regions showing prominent NF-H/M somatic staining in amygdalar neurons of PS cKO, PS1 cKO;Tau and PS cKO;Tau mice. Scale bar = 100 m. **B**, Representative average infrared spectra of WT and PS cKO;Tau mice in the retrosplenial cortex (RSC) and corpus callosum (CC) indicating the main absorptions peaks with their corresponding chemical functional groups. Abbreviation: a.u.: arbitrary units. **C**, Synchrotron-based µFTIR analysis of the RSC and CC of WT and PS cKO;Tau mice at 6 months of age showing lipid oxidation by C=CH/CH2 (d^2^A_3014_/d^2^A_2921_) and C=O/CH2 (d^2^A_1741_/d^2^A_2921_) ratios, and protein/lipid amount by Amide I/CH2 (d^2^A_1635_+1656/d^2^A_2921_) ratio. Values represent the minimum, the maximum and the median of the average of 100 spectra/mouse (n = 4 mice/group).

**Additional file 3**. Phosphorylated tau is present in synaptosomes of Tau transgenic mice. **A**, Western blot images of phosphorylated tau and synaptic proteins in lysates and purified synaptosomes and presynaptic and postsynaptic fractions from hippocampus of 6-9 month-old WT and Tau mice. **B-D**, Quantitative analysis of phosphorylated Ser396/404 tau (PHF-1), PSD95, CRTC1, and synaptophysin in synaptosomal (**B**), presynaptic (**C**) and postsynaptic (**D**) fractions of WT and Tau mice. Values represent mean fold ± s.e.m. (n = 3 mice/group). Statistical analysis was determined by unpaired student’s *t*-test. *P < 0.05.

## Data Availability

Data and materials, including specific experimental protocol information, are available under request.
