# Supplementary figures and images for: Loss of presenilin function enhances tau phosphorylation and aggregation in mice
Source: Acta Neuropathol Commun. 2021 Sep 30;9:162. doi: 10.1186/s40478-021-01259-7 (PMC8482568; doi:10.1186/s40478-021-01259-7)

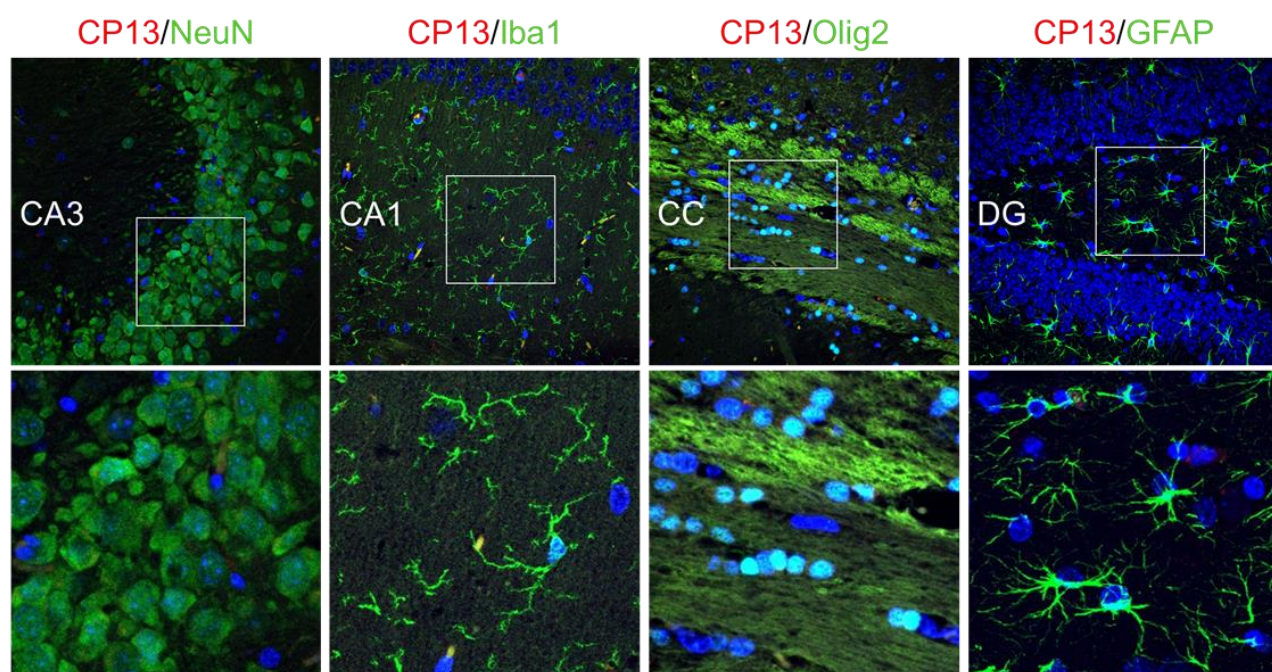

Supplementary Figure 1

Supplement: Supplementary file 1 — Additional file 1. CP13 immunostaining in control mice. CP13 staining (red) is barely detected in neurons (NeuN; green), microglia (Iba1; green), oligodendrocytes (Olig2; green) and astrocytes (GFAP; green) in brain sections of control (WT) mice. Insets: magnified images of the indicated selected regions of CA3, CA1, CC and DG (upper images) are shown at the bottom. Abbreviations: CA1/CA3 hippocampus; DG: dentate gyrus; CC, corpus callosum. [file 40478_2021_1259_MOESM1_ESM.pdf]

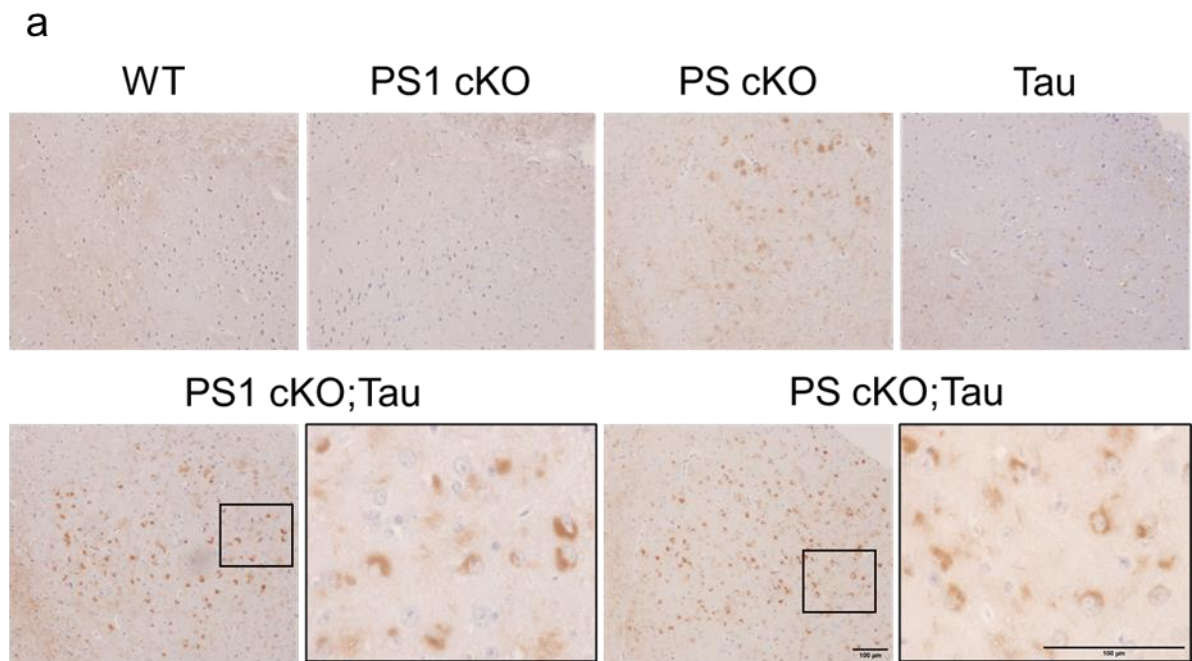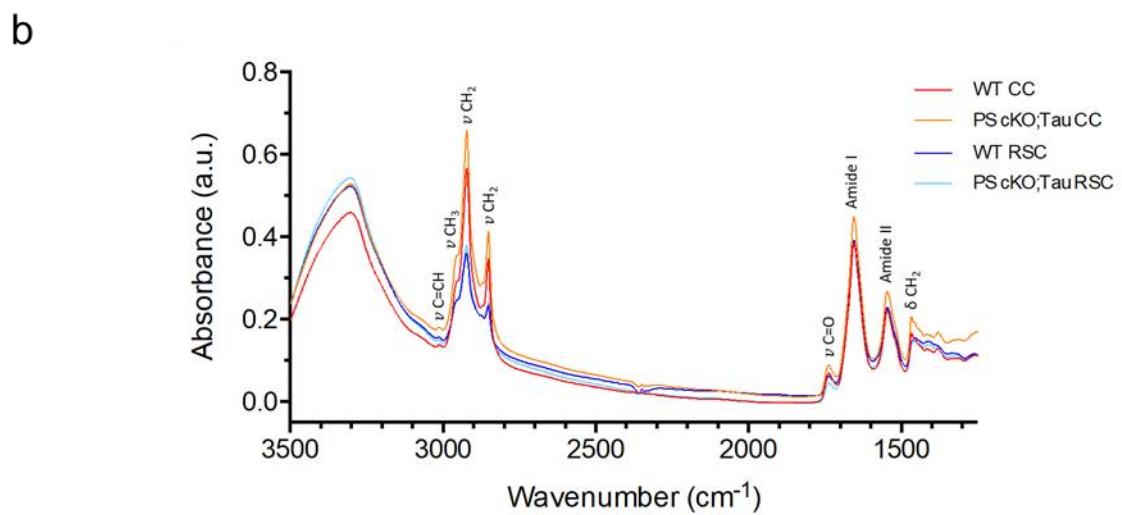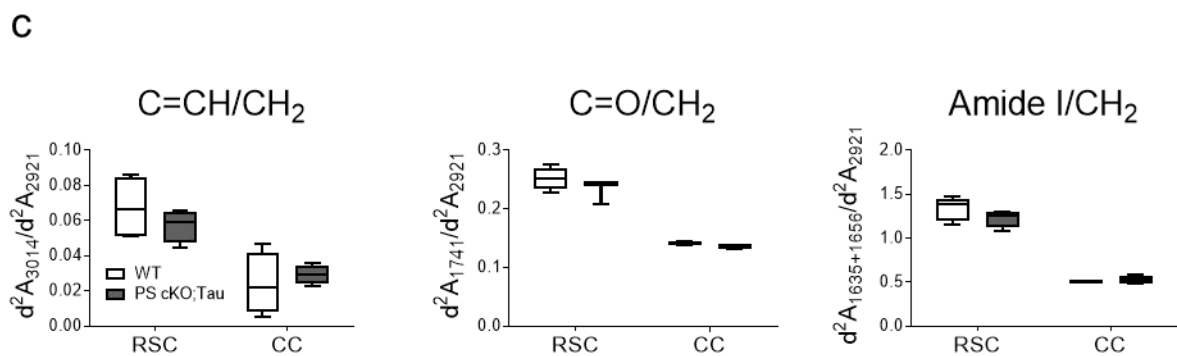

Supplementary Figure 2

Supplement: Supplementary file 2 — Additional file 2. NF staining and synchrotron-based µFTIR analysis of PS-deficient Tau mice. A, Immunohistological images of SMI312 (NF-H/M) in the basolateral amygdala of WT, PS1 cKO, PS cKO, Tau, PS1 cKO;Tau and PS cKO;Tau mice at 6 months of age. Insets: magnified images of the indicated left square regions showing prominent NF-H/M somatic staining in amygdalar neurons of PS cKO, PS1 cKO;Tau and PS cKO;Tau mice. Scale bar = 100 m. B, Representative average infrared spectra of WT and PS cKO;Tau mice in the retrosplenial cortex (RSC) and corpus callosum (CC) indicating the main absorptions peaks with their corresponding chemical functional groups. Abbreviation: a.u.: arbitrary units. C, Synchrotron-based µFTIR analysis of the RSC and CC of WT and PS cKO;Tau mice at 6 months of age showing lipid oxidation by C=CH/CH2 (d2A3014/d2A2921) and C=O/CH2 (d2A1741/d2A2921) ratios, and protein/lipid amount by Amide I/CH2 (d2A1635+1656/d2A2921) ratio. Values represent the minimum, the maximum and the median of the average of 100 spectra/mouse (n = 4 mice/group). [file 40478_2021_1259_MOESM2_ESM.pdf]

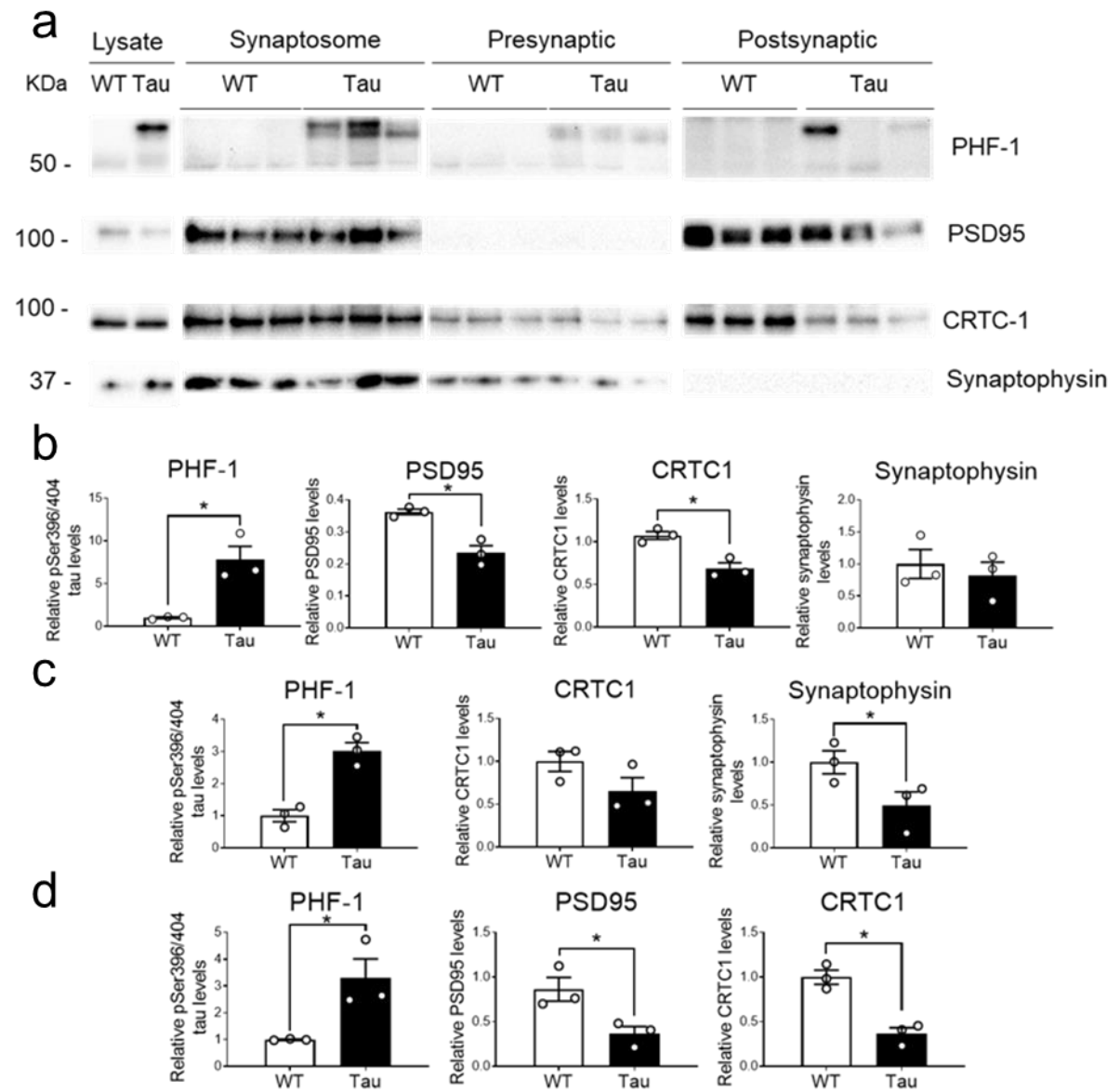

Supplementary Figure 3

Supplement: Supplementary file 3 — Additional file 3. Phosphorylated tau is present in synaptosomes of Tau transgenic mice. A, Western blot images of phosphorylated tau and synaptic proteins in lysates and purified synaptosomes and presynaptic and postsynaptic fractions from hippocampus of 6-9 month-old WT and Tau mice. B-D, Quantitative analysis of phosphorylated Ser396/404 tau (PHF-1), PSD95, CRTC1, and synaptophysin in synaptosomal (B), presynaptic (C) and postsynaptic (D) fractions of WT and Tau mice. Values represent mean fold ± s.e.m. (n = 3 mice/group). Statistical analysis was determined by unpaired student’s t-test. *P < 0.05. [file 40478_2021_1259_MOESM3_ESM.pdf]
